# Human Health

**Published:** 1883-02

**Authors:** 


### HUMAN HEALTH.

The most forcible argument, is an appeal to the pocket; before it, independence crouches; prejudices vanish; the pride of consistency veils its face; and the warm love of kindred, and party, and religion congeals to a stone; it teaches men to observe, and compels them to the exercise of common sense.

A New York drayman or hack driver, considers his horse a part and parcel of himself, and the moment his animal ceases motion in cold weather, that moment he covers him with a blanket. Why this care? He knows that if neglected, the horse will take cold, and that in a day or two, he will most probably die of some form of inflammation about the lungs; yet multitudes of people perish every year, from being cooled off too quick after exercising.

More people die prematurely from want of care in any given year, than perish by plague, famine, pestilence and war.

The Duke of Wellington died of an over hearty meal of venison in November.

General Taylor was taken from the White House to the grave, by a bowl of fruits and iced milk, on a fourth of July.

And we need not enumerate the countless multitudes, who are gradually led to the grave, by the various intoxications of alcohol, opium and tobacco, from ignorance of the fact, that the appetite for them feeds upon itself, and grows by its indulgence.

No reformation can be relied on, which is not founded on intelligence, associated with a stern religious principle. Hence, it is not the doctor who is to renovate human health, and build up a new physical constitution for coming generations. All radical reforms aim at prevention, rather than rectification. To prevent a man from getting sick, is a more glorious mission than to cure him. The parent, the teacher, the minister, these are the parties who are to co-operate in raising sons and daughters of robust health, and cultivated intellect, and educated consciences, to occupy the responsible positions of a coming age.

It is a good omen, that intelligent, reflecting and humane teachers in different parts of the country, are beginning to make personal health, one of the branches of an elementary education. Is it not wonderful that more efficient steps have not been taken in that direction, long ago.

